# Ribosomal control in RNA virus-infected cells

**DOI:** 10.3389/fmicb.2022.1026887

**Published:** 2022-11-07

**Authors:** Xiao Wang, Jie Zhu, Da Zhang, Guangqing Liu

**Affiliations:** Shanghai Veterinary Research Institute, Chinese Academy of Agricultural Sciences, Shanghai, China

**Keywords:** ribosome, translation, RNA viruses, heterogeneous ribosomes, protein

## Abstract

Viruses are strictly intracellular parasites requiring host cellular functions to complete their reproduction cycle involving virus infection of host cell, viral genome replication, viral protein translation, and virion release. Ribosomes are protein synthesis factories in cells, and viruses need to manipulate ribosomes to complete their protein synthesis. Viruses use translation initiation factors through their own RNA structures or cap structures, thereby inducing ribosomes to synthesize viral proteins. Viruses also affect ribosome production and the assembly of mature ribosomes, and regulate the recognition of mRNA by ribosomes, thereby promoting viral protein synthesis and inhibiting the synthesis of host antiviral immune proteins. Here, we review the remarkable mechanisms used by RNA viruses to regulate ribosomes, in particular, the mechanisms by which RNA viruses induce the formation of specific heterogeneous ribosomes required for viral protein translation. This review provides valuable insights into the control of viral infection and diseases from the perspective of viral protein synthesis.

## Introduction

Viruses are intracellular parasitic organisms with infectious potential and are divided into two categories, namely DNA viruses and RNA viruses, according to their genomic characteristics. In recent years, RNA viruses have attracted the increasing attention of researchers because of their high adaptability, high variability, and strong pathogenicity (Colson et al., 2022; Liu et al., 2022; Tomar et al., 2022). Depending on their genomic characteristics, RNA viruses can be classified as double-stranded RNA viruses, positive-stranded RNA viruses and negative-stranded RNA viruses. Of course, the life cycle of different RNA viruses in the cell also differs. However, viruses invade the host cell by binding to receptors on the cell membrane, releasing the viral genome into the cell, subsequently initiating the translation of viral proteins, and performing genome replication, viral assembly, and viral budding and release, thereby completing the viral replication cycle. Viruses complete each step of their life cycle through an intricate interaction with the host cells. Because the viral genome is small and does not have a translation system for coded proteins, all viruses use the host protein synthetic machinery to translate viral mRNAs. To form mature ribosomes and initiate the translation of viral proteins, viruses recruit the host’s protein translation initiation factors through genomic RNA 5′-UTR-specific structures or covalently bound viral proteins to generate translation initiation complexes that subsequently bind to both the small and large subunits of the ribosomes (Lopez-Lastra et al., 2010). With the evolution of viruses, different viral species have adopted different mechanisms for recruiting translation initiation factors such as the classical cap-dependent and cap-independent types (Internal Ribosome Entry Site [IRES], etc.) (Vallejos et al., 2010; Filbin et al., 2013; Olivares et al., 2014). In addition, during the life cycle which are as follows. Viruses usurp the host translation machinery, encode multifunctional proteins to regulate gene expression, use overlapping open reading frames to store viral genetic information, or use a single messenger RNA to synthesize multiple proteins, all of these processes require a series of sophisticated strategies to evade host immune surveillance (Meyers, 2003; Gaucherand et al., 2019; Liu et al., 2019). Presently, the mechanism of viral protein translation initiation is well understood. However, the mechanism by which viral mRNA forms the translation initiation complex and then recruits ribosomal subunits to form mature ribosomes required for viral protein translation is unclear.

The ribosome is the host protein synthesis factory, and it is formed by the assembly of rRNA, the 40S ribosomal subunit, and the 60S ribosomal subunit. There are more than 80 known ribosomal proteins (RPs), the 40S subunit comprises about 30 proteins, while the 60S subunit contains 50 proteins, but it has been found that not all RPs are involved in the formation of mature ribosomes. However, not all RPs are involved in the formation of mature ribosomes (Hamidi et al., 2018). Therefore, the size and composition of ribosomes in different microenvironments may vary correspondingly. Around the 1980s, it was found that different mRNAs are translated by specific ribosomes (Hui and de Boer, 1987; Ferretti et al., 2017). Ribosomal heterogeneity refers to changes in the stoichiometry of rRNAs and RPs during ribosome generation as well as post-transcriptional rRNA and post-translational RP modifications that result in the incorrect expression of some genes (Genuth and Barna, 2018; Cheng et al., 2019; Emmott et al., 2019). Defects or accumulation of substances required for body metabolism can lead to ribosomopathies such as Diamond–Blackfan anemia (DBA), some cancers, some neurodevelopmental disorders such as autism spectrum disorders and microcephaly are associated with heterogeneous ribosomes or mutated RPs (Segev and Gerst, 2018). Recent evidence suggests that viral infection induces heterogeneous ribosome production, leading to changes in ribosome conformation and composition (DiGiuseppe et al., 2020).

In this review, we focus on the regulation of the host ribosome following viral invasion of the host cell. Including how viruses shut down ribosomal translation of the host genome and how viruses use the host ribosome to preferentially translate the viral genome.

## RNA viruses shut down host protein translation by regulating ribosomes

Viral infection is a dynamic process in which viruses and organisms compete with each other. Depending on the mechanism of infection, viruses can be classified as those that cause cell-killing, stable, and integrated infections (Maree et al., 2016). Compared to viruses that cause stable and integrated infection of the host, cell-killing viruses encode early viral proteins that capture the synthesis machinery of the host cell for their use to inhibit or even shut down the synthesis of host RNA and proteins (Curran and Kolakofsky, 1989; Vidal et al., 1990; Boeck et al., 1992; Chenik et al., 1995). Through this approach, viruses can disrupt normal cellular metabolism and ultimately lead to cell death. Hence, this section focuses on two aspects of RNA viruses that induce ribosomes to shut down the translation of host proteins: inhibition of host mRNA recognition and prevention of ribosome subunit assembly.

### RNA viruses inhibit the recognition of host mRNA by ribosomes

During viral infection, the virus regulates the expression of host proteins through the control of ribosomes. The main pathways include remodeling the host mRNA pool and inhibiting the recognition of host mRNA by ribosomes. Currently, some viruses can remodel the host mRNA library by encoding viral proteins that interfere with host pre-mRNA synthesis, induce host mRNA degradation, or block host mRNA nuclear export pathway. Among these approaches, interference with host mRNA synthesis is mainly achieved by strategies such as inhibition of pre-mRNA splicing by the incorporation of poly(A) or direct inhibition of pre-mRNA polyadenylation. For example, the Vpr protein of human immunodeficiency virus (HIV) was identified to inhibit pre-mRNA splicing, and the NS1 protein of avian influenza virus (AIV) inhibits pre-mRNA splicing and polyadenylation (Nemeroff et al., 1998; Hashizume et al., 2007; Nacken et al., 2021). Moreover, the NSP1 protein of severe acute respiratory syndrome coronavirus 2 (SARS-CoV-2), the polymerase of feline calicivirus (FCV), and the PA-X protein of AIV were confirmed to degrade host mRNA (DuBois et al., 2012; Khaperskyy et al., 2016; Finkel et al., 2021; Wu et al., 2021). The inhibition of nuclear export of host mRNA mainly occurs by encoding viral proteins that interfere with the normal biological role of nuclear pore proteins, thereby inhibiting the recognition of host mRNA by the ribosome and facilitating the expression and inheritance of viral proteins such as the L protein of Theiler’s murine encephalomyelitis virus (TMEV), 2A protein of poliovirus (PV), M protein of vesicular stomatitis virus (VSV), and 3C protein of human rhinovirus (HRV) (von Kobbe et al., 2000; Castello et al., 2009; Walker et al., 2013; Masaki et al., 2019).

In recent years, some viruses were found to prevent ribosomes from recognizing host mRNA, resulting in the inhibition of normal cellular physiological functions and providing a more supportive environment for virus proliferation. In studies of Severe Acute Respiratory Syndrome Coronavirus 2 (SARS-CoV-2), which causes the worldwide coronavirus disease 2019 (COVID-19) pandemic affecting millions of people, it was found that the viral non-structural protein 1 (NSP1) encoding the virus early in infection binds with high affinity to the ribosomal mRNA entry channel, preventing recognition of host and viral mRNA by the ribosome (Tidu et al., 2020). The interaction between NSP1 and RPs RPS3, RPS2 and rRNA h18 relies mainly on electrostatic and hydrophobic forces (Yuan et al., 2020). The viral genome hijacking ribosome mainly uses 5′ end stem-loop structure to interact with NSP1, regulating the binding between NSP1 and 40S and exposing the mRNA entry channel to complete the translation of the viral genome (Tidu et al., 2020). Such fine-grained regulatory roles between the host ribosome and SARS-CoV-2 should leave much to be investigated. Another example is about the level of host protein expression was significantly suppressed in the late stages of HIV-1 infection due to the high expression of host RNA decapping enzyme (DDX3) (Labaronne et al., 2022). DDX3 has been found to be hijacked by a variety of RNA viruses, facilitating viral transcription, nuclear export, translation and assembly of viral particles, while threatening the innate immune response (Valiente-Echeverria et al., 2015). It follows that different strategies can be used to inhibit translation of host proteins by the ribosome, regardless of the type of viral infection.

### RNA viruses affect ribosomal small and large subunit assembly

Ribosome biogenesis is primarily in the nucleolus, where rRNA precursors (pre-rRNA) are processed into mature molecules with mature RPs that assemble into 40S ribosomal subunits and 60S ribosomal subunits for release into the cytoplasm. The 40S ribosomal subunit forms a 48S pre-initiation complex with the translation initiation complex and mRNA, which subsequently recruits the 60S ribosomal subunit to eventually form the translationally functional 80S ribosome. Viruses cannot carry their protein synthesis machinery and need to manipulate ribosomes to complete their protein translation. Hence, the viral genome competitively utilizes ribosomes with host mRNA. Viruses have evolved mechanisms to influence the assembly and maturation of ribosomes.

Some viral proteins interact directly with the host 40S or 60S, thereby preventing the formation of the 43S initiation complex and the assembly of the 60S and 40S subunits. For example, SARS-CoV NSP1 protein interacts with the 40S ribosomal subunit to inhibit the formation of the 80S ribosome (Kamitani et al., 2009; Lokugamage et al., 2012). Notably, the effects of NSP1 encoded by different coronaviruses on the ribosome are different. For example, the NSP1 protein encoded by Middle East respiratory syndrome coronavirus (MERS-CoV) binds with different stability and intracellular localization to the 40S ribosomal subunit as compared to the NSP1 protein encoded by SARS-CoV-2 (Lokugamage et al., 2015). This suggests that the degree of selective inhibition of host protein translation through the strategy of blocking ribosomal mRNA entry into the channel by the binding of NSP1 to the 40S subunit varies with different coronaviruses. Discovery of similar mechanisms is relatively rare, mainly due to the fact that translation of the viral genome requires an intact ribosome to synthesize viral proteins.

The modification and splicing of eukaryotic translation initiation factors required for ribosome assembly by viruses lead to ribosome-mediated translation barriers. Most RNA viruses inhibit host protein translation by preventing the binding of ribosomal subunits through strategies such as promoting phosphorylation or dephosphorylation modification of translation initiation factors, cleaving eukaryotic translation initiation factors, or cleaving poly(A)-PABP (Lamphear et al., 1995; Gingras et al., 1996; Gradi et al., 1998; Kerekatte et al., 1999; Kuyumcu-Martinez et al., 2004; Willcocks et al., 2004; de Breyne et al., 2008; Rojas et al., 2010). The coat proteins encoded by neuro necrosis virus (NNV) manipulate PABP from the cytoplasm of the host cell into the nucleus to degrade it by ubiquitination (Cheng et al., 2021). Thus, viruses inhibit the expression of the host protein but do not affect the expression of the viral protein.

In contrast, viruses can use the diversity of genomes and the expression of viral proteins to promote the assembly of ribosomal subunits and facilitate the formation of heterogeneous ribosomes to selectively translate the viral genome (Lago et al., 2017). The ribosomal protein RPL13 interacts with the IRES element of the Seneca Valley virus (SVV) and classical swine fever virus (CSFV) to manipulate ribosomes to translate viral proteins (Han et al., 2020). At present, it has been confirmed that the IRES structure of Foot and Mouth Disease Virus (FMDV) utilizes the interaction between RPL13 and helicase DDX3 to promote the assembly of 80S ribosomes and then translate viral protein (Han et al., 2020). This approach was also found in other picornaviruses such as coxsackievirus (CBV). However, DDX3 cannot contribute to the translation of other RNA viruses that do not contain IRES elements, such as VSV and measles virus (MV) (Quaranta et al., 2020). Together, these findings demonstrate the IRES element of the viral genome could manipulate the host’s antiviral factor to inhibit the host natural immune response and thereby facilitate viral protein translation.

**Figure 1:**
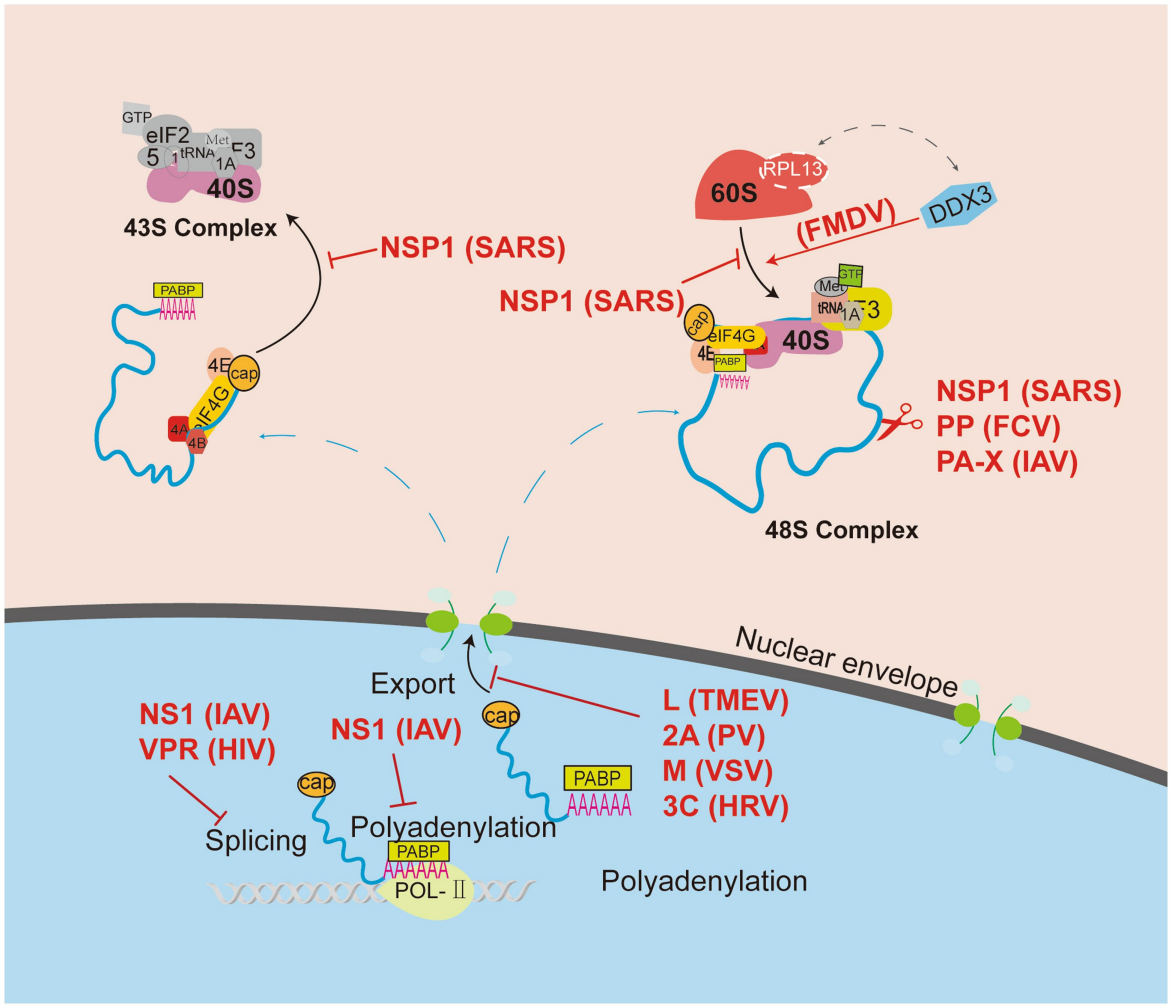
Viruses manipulate ribosomes to shut down host protein translation. Cellular mRNA biogenesis begins in the nucleus, and viruses use various mechanisms to block the translation of host mRNA, including remodeling of the host mRNA library, inhibiting host mRNA transport from the nucleus, and blocking ribosomal recruitment of eukaryotic translation initiation factor (eIF). Consequently, viruses can block ribosomal mRNA entry channels or inhibit the binding of ribosomal size subunits to prevent the translation of host proteins.

**Figure 2:**
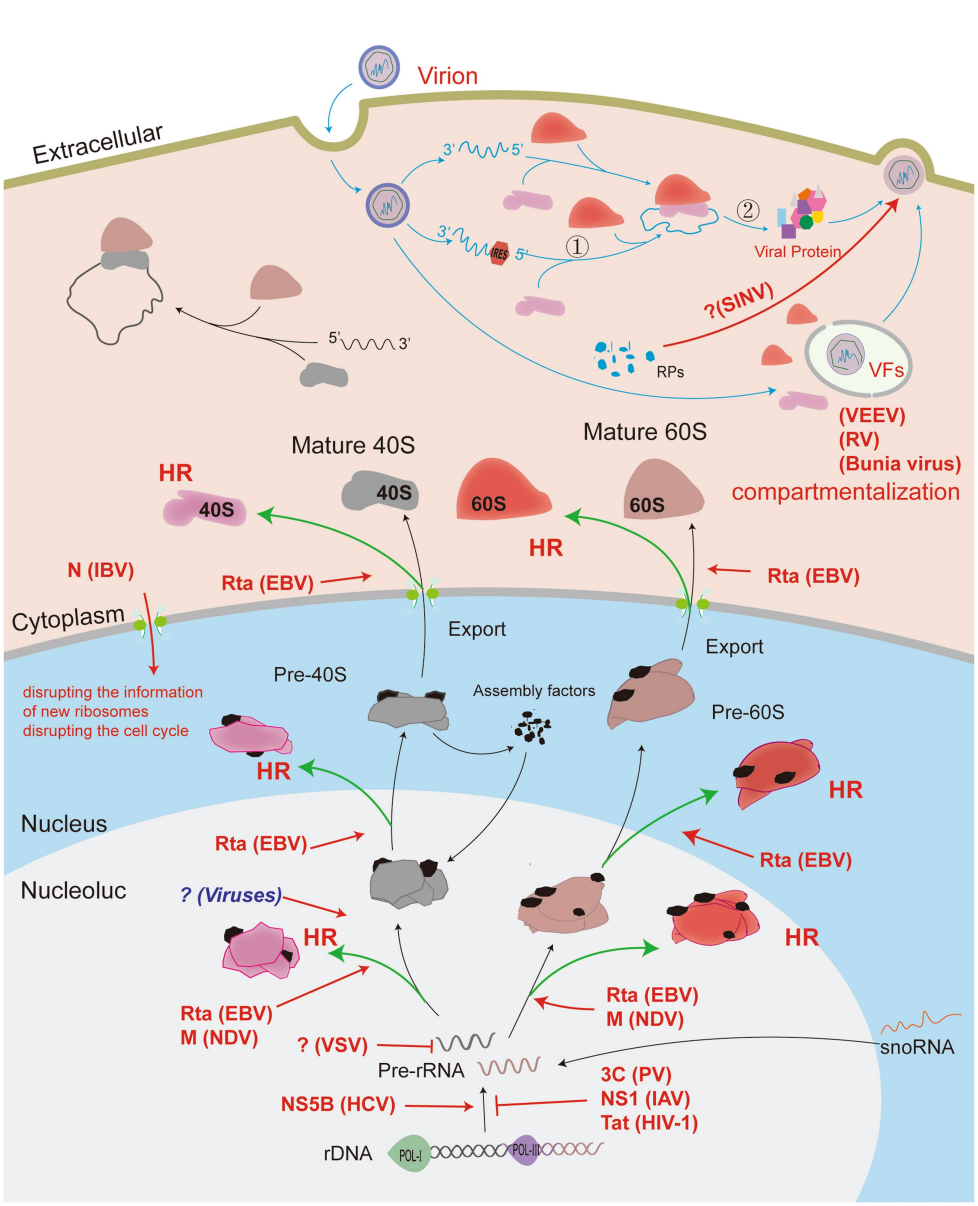
Viruses manipulate ribosomes to complete the translation of viral proteins. Viruses invading host cells can manipulate ribosomes to initiate the translation of viral proteins by modulating their own genome structure. The viral genome can also regulate the distribution of host ribosomal compartments or manipulate host ribosomal components to assemble viral particles that facilitate viral protein translation. Ribosome biogenesis begins in the nucleolus, and RNA virus infection causes nucleolus stress, with viral proteins acting on the different functional regions of the nucleolus to induce heterogeneous ribosome biosynthesis (HR). The effect of the virus on the host (red arrow); normal host physiological processes (black arrow); the process of virus proliferation in the cell (blue arrow); the virus-mediated production of heterogeneous ribosomes (green arrow). Process ① represents the recruitment of ribosomes by viruses using a cap-independent structure. Process ② indicates the translation process wherein viruses can use the specific structure of their own mRNA to reinitiate downstream viral proteins.

In summary, RNA viruses manipulate the functional ribosome to translate the viral mRNA. However, the biosynthesis and assembly mechanism of ribosomes are very complex and elusive. As shown in Figures 1, 2, RNA viruses use different strategies to control the ribosome, such as inhibiting of ribosome recognizing host genome and preventing the assembly of large and small ribosomal subunits. The mechanisms by which RNA viruses affect ribosome assembly remain unclear. For example, it is unknown why viruses affect the expression of some host proteins but not all. Some studies have shown that the ribosomal components are not fully involved in the assembly of ribosomes, and the ribosome structures in different cell regions are significantly different (Shi and Barna, 2015; Luan et al., 2022). These findings indicate that the ribosome is composed of a core region and various genetically adapted RPs (Ngoveni et al., 2019; Patino-Galindo et al., 2021). Therefore, the effect of heterogeneous ribosomes on viral protein translation deserves further study.

## Viruses hijack ribosomes to complete viral protein translation

Viral infection induces a comprehensive stress response in cells that stimulates the phosphorylation of eIF2α and inhibits viral proliferation (Huang et al., 2017). To defend against host antiviral responses, some viruses have evolved cap-dependent translation initiation mechanisms. For example, some viruses adopt a strategy that is independent of the translation initiation factor eIF2 during the translation process (Garaigorta and Chisari, 2009; Roth et al., 2017). Regardless of this mechanism, the ribosome is also the key machine for viral protein translation. Here, we review strategies for the binding of viral RNA to ribosomes to initiate protein translation and the effects of viral infection on the assembly and conformation of the ribosomal size subunit (as shown in Figure 2).

### Strategies for viral RNA bind to ribosomes

Mutation or recombination of the viral genome can increase its diversity, thereby allowing viruses to easily adapt to a new host and to mediate immune escape and exhibit resistance to gene therapy (Pilipenko et al., 1992; Miras et al., 2014; Xiao et al., 2016; Ngoveni et al., 2019; Patino-Galindo et al., 2021). This evolutionary pattern makes the viral genome more prone to prey on ribosomes. Viruses have evolved various strategies to initiate viral protein translation, such as cap-dependent, cap-independent, and IRES-dependent approaches.

#### Viral IRES element manipulates ribosome

Discovered in 1988 by Jang and Pelletier et al., IRES is a secondary structure of the untranslated region (UTR) at the 5′ end of PV mRNA that binds directly to 40S and remodels the ribosome structure to form a heterogeneous ribosome that preferentially translates the viral genome (Jang et al., 1988; Pelletier and Sonenberg, 1988; Jobe et al., 2019). Notably, it has been demonstrated that the HCV IRES structure can directly hijack the translating 80S ribosome, but the subsequent mechanism of action needs further investigation (Yokoyama et al., 2019). Currently, IRES structures have been identified in picornaviruses, flaviviruses, retroviruses, and some cellular mRNAs, but IRES elements in these RNA viruses lack conserved features (Martinez-Salas et al., 2017). According to the secondary structure of IRES, it is mainly divided into five types (I–V). Each type of IRES requires different translation initiation factors to initiate translation, and the pattern of binding of each type of IRES to ribosomes is also different (as shown in Table 1; Martinez-Salas et al., 2017).

**Table 1:** Characteristics and classification of IRES of RNA viruses.

IRES	Structure features	Required initiation factors	Examples	References
Type I	Contains five stem-loop structural domains II, III, IV, V, VI and ribosome recognition of the IRES structure requires scanning of the start codon	eIF1A, eIF2, eIF3, eIF4A, eIF4B, eIF4G	PV; Enterovirus 71 (EV 71); Coxsackievirus type B3 (CV B3); HRV	Walter et al. (2002); Thompson and Sarnow (2003); Bhattacharyya et al. (2008); Sean et al. (2008); Tolbert et al. (2017); Beckham et al. (2020); LaFontaine et al. (2020)
Type II	Contains structural domains 2–5 (also known as H, I, J, K, L), multiple start codons and the start codon binds directly to the ribosome to initiate translation	eIF2, eIF3, eIF4A, eIF4B, eIF4G	EMCV; FMDV; Equine rhinitis B virus (ERBV)	Davies and Kaufman (1992); Hinton and Crabb (2001); Chamond et al. (2014); Quaranta et al. (2020)
Type III	Contains three structural domains, III, IV and V, and structural domains III and IV are required for viral translation	eIF2, eIF3, eIF4A, eIF4B, eIF4G/eIF4F	HAV	McKnight and Lemon (2018)
Type IV (HCV-like)	Contains three structural domains II, III and IV, with structural domain IIId2 being the required structure for IRES activity	eIF2, eIF3	Seneca Valley virus (SVV); Porcine teschovirus (PTV)	Bakhshesh et al. (2008); Willcocks et al. (2017)
Type V	Contains structural domains I, J, K, L, with the start codon located enclosed in the hairpin structure	eIF2, eIF3, eIF4A, eIF4B, eIF4G, ITAFs (DHX29)	Aichivirus	Bakhshesh et al. (2008); Yu et al. (2011); Sweeney et al. (2012)
HCV IRES	Flexible structure formed by three rigid structural domains II, III and IV, dynamically bound to the ribosome	eIF2, eIF3	HCV; CSF	Yu et al. (2005); Yamamoto et al. (2015); Ullah et al. (2019)
IGR IRES	Consists of a triplet of pseudoknots (PKI, II and III) that mimic tRNA at the ribosomal p site to initiate translation	None	Cricket paralysis virus (CrPV); Israeli Acute Paralysis Virus (IAPV)	Wilson et al. (2000); Ullah et al. (2019); Walters et al. (2020)
Retrovirus IRES	Includes three core structural domains, the primer binding site (PBS), the dimerization initiation site (DIS) and the splice donor, mainly located in the 5’ UTR and Gag proteins	eIF4A, eIF5A	HIV-I; Human T-lymphotropic virus 1 (HTLV-1); MMTV	Caceres et al. (2016); Carvajal et al. (2016); Ullah et al. (2019)

IGR IRES of double-stranded viruses represent the simplest class of IRES (Roberts and Groppelli, 2009; Kerr et al., 2015; Gross et al., 2017). Each ORF encoded by the virus has unique IRES structure (Roberts and Groppelli, 2009; Kerr et al., 2015; Gross et al., 2017). However, many other IRES structures are challenging to categorize because of their diversity, such as IRES structures in SARS-CoV-2, alphavirus, infectious pancreatic necrosis virus (IPNV), drosophila C virus (DCV), dengue fever virus (DENV), and calicivirus (Kamrud et al., 2007; Landry et al., 2009; Rivas-Aravena et al., 2017; Fernandez-Garcia et al., 2020; Arhab et al., 2022; Slobodin et al., 2022). Notably, viral IRES elements are expressed in heterologous viral genomes in hosts with mixed infectious viruses (Arhab et al., 2020). In the case of avian calicivirus and picornavirus co-infection, the 5’-UTR of avian calicivirus was found to contain a conformation consistent with the 5’-UTR secondary structure of picornavirus (IRES type V) (Arhab et al., 2022). These discoveries of IRES structure may be due to genetic recombination with other viruses and reflects the importance of analyzing the interaction of the ribosome with the viral RNA genome to resolve the mechanism of viral protein synthesis.

#### Viral strategies for manipulating the ribosome to translate viral polycistronic ORFs

Eukaryotic mRNAs are usually monocistronic, while some viral mRNAs are polycistronic. Thus, in order to survive and reproduce in the host cell, viruses have a superior means of solving the problems of time, space and quality of genetic information expression than cells. The strategies include leaky scanning, translation termination re-initiation, ribosomal shunting, ribosome hopping, and frameshifting. As summarized in Table 2, these re-initiation strategies vary in their approach and characteristics.

**Table 2:** Different translation initiation strategies of RNA viruses.

**Strategies**	**Approach to strategy**	**Examples**	**References**
Leaky scanning	The way ribosomes skip translation of weak start codon codons on mRNA during scanning of the 5′ end of the viral genome	Bovine Coronavirus (BCV); Rhabdovirus; RV	Chenik et al. (1995); Senanayake and Brian (1997); Racine and Duncan (2010); Smirnova et al. (2015)
Ribosomal termination-reinitiation	Multiple cis-trans viral genomes, in which the ribosome moves to the upstream ORF termination codon after the 40S ribosomal subunit still continues to move to the downstream ORF in the presence of the upstream ribosome binding site; reinitiation of translation.	RHDV; FCV; Norovirus; (IAV)	Meyers (2007); Habeta et al. (2014); Luttermann and Meyers (2014); Zinoviev et al. (2015); Wennesz et al. (2019)
Shunt	The 40S ribosomal subunit interacts with the 5′ cap structure of the mRNA in a scan that bypasses the larger stem-loop structure and begins a short distance downstream of the initiation site. Primarily, this allows translation initiation to be located close to the internal codon of the ribosomal receptor sequence.	Sendai virus; Avian reovirus; HIV-1, Duck hepatitis B virus (DHBV); Foamy virus	de Breyne et al. (2003); Schepetilnikov et al. (2009); Cao and Tavis (2011); Rethwilm and Bodem (2013)
Ribosomal hopping	The way in which the ribosome recognises that the viral genome encodes a specific sequence and then prevents the formation of an ester bond between the polypeptide chain and the next amino acid, while the ribosome continues to translate new viral mRNA along that chain.	FMDV; Duck hepatitis A virus-1 (DHAV)	Luke et al. (2008); Minskaia and Ryan (2013); Kjaer and Belsham (2018); Yang et al. (2018)
Ribosomal frameshifting (PRF)	In overlapping ORFs, ribosomal recognition of specific sequences in the mRNA results in a ribosomal shift to reinitiate the expression of the downstream ORF of initiation	PRRSV; SimianHemorrhagicFeverVirus (SHFV)	Namy et al. (2006); Li et al. (2019); Yang et al. (2020); Bhatt et al. (2021); Napthine et al. (2021)

Viral genomes take advantage of diverse translation strategies not only to facilitate the production of two or more proteins but also to regulate the ratio of viral proteins generated. Viruses regulate gene expression by modulating the arrangement of nucleotides on mRNA, for example by forming specific Kozak sequences or relatively complex secondary structures such as DPL sequences, TURBS sequences and 2A sequences, which affect the rate and mode of translation of the ribosome. As found in both Sendai and parainfluenza viruses, the viral genome can cleverly hijack ribosomal initiation for translation by using different initiation codons through nucleotide changes at specific sites of mRNA (Curran and Kolakofsky, 1989; Vidal et al., 1990; Boeck et al., 1992). Notably, the ribosomal “skipping” occurs inside the ribosome with little influence from external factors. The nucleotides of the viral protein are very delicately arranged and facilitate the stable expression of viral genes by regulating the movement of ribosomes on mRNA. Therefore, identifying the specific roles of ribosome components in the movement of RNA virus genomes is particularly important for the assembly mechanism of ribosomes required for viral protein translation.

#### Viruses regulating ribosome distribution or manipulating ribosomal proteins

Viral infection induces ribosome aggregation. RNA viruses induce ribosomal localization to recruit viral mRNA. In eukaryotes, some transcripts could be assembled into specific RNA condensates to form ribonucleoprotein (RNP) particles (Kachaev et al., 2021). These particles are transported by motor proteins to sites where translation is active, thus resulting in areas of liquid–liquid phase separation in the cytoplasm (Kachaev et al., 2021). Some RNA viruses, such as Zika virus (ZIKV), DENV, and hepatitis C virus (HCV) infect host cells to stimulate specific intracellular structures to form virus factories (VFs) (Paul et al., 2014; Reid et al., 2018; Mohd Ropidi et al., 2020). VFs could facilitate the replication and assembly of viral genomes, but additional studies are required to determine whether VFs recruit ribosomes to mediate the generation of heterogeneous ribosomes (Lashkevich and Dmitriev, 2021). As early as the 1970s, ribosomal aggregates in the VF formed early in Venezuelan equine encephalomyelitis virus (VEEV) infection were identified by electron microscopy (Bykovsky et al., 1969). It is worth mentioning that ribosomes and translation initiation factors are clustered around VFs formed in the bunyavirus and reovirus infection (Matsumoto et al., 2012; Desmet et al., 2014). In addition, Reoviruses infect cells mainly by regulating the distribution of translation initiation factors, but not by promoting the production of translation initiation factors. Taken together, these studies suggest that viral infection induces intracellular compartmentalization of the host translational machinery and replicative components.

Ribosome-associated proteins are involved in viral particle assembly. Different from the machines by viral genome, Sindbis virus (SINV) infects hosts with viral particles that contain host-derived ribosomal components, and SINV^Heavy^ is more infectious than SINV^Light^ because of its ribosomal component (Sokoloski et al., 2013; Mackenzie-Liu et al., 2018). SINV^Heavy^ particles are found only in vertebrates, whereas SINV^Light^ particles are found in invertebrate blood. The intermediate host mosquito can absorb only SINV^Light^ particles, but the virus particles undergo proliferative replication in the mosquito to produce SINV^Heavy^ virus particles to reinfect vertebrates (Sokoloski et al., 2013; Mackenzie-Liu et al., 2018). This mechanism suggests that the virus manipulates the ribosomal component of the host as it proliferates in the host, but whether this mechanism contributes to the assembly of ribosomal subunits during the next round of viral genome translation needs to be further explored.

### RNA virus induces heterogeneous ribosome production

With the development of cryo-electron microscopy, the delicate structure of the ribosome is gradually being revealed. Numerous studies have found that viral infection affects ribosome biogenesis, modification of ribosomal components, and changes in ribosome conformation.

#### Viral infection affects ribosome biogenesis

The biosynthesis of ribosomes occurs mainly in the nucleolus, and disturbances in the process at any point can potentially lead to nucleolar stress, is also known ribosomal stress (Bianco and Mohr, 2019; Iarovaia et al., 2021; Luan et al., 2022). To manage this stressful state, cells cease the transcription of ribosomal genes (Iarovaia et al., 2021). Hence, the intensity of ribosome biogenesis is directly dependent on cellular demand and the strength of cell growth (Iarovaia et al., 2021).

Some RNA virus infections are accompanied by viral proteins that need to be transported to the nucleolus and possibly affect ribosome biogenesis. The HCV NS5B protein and core protein upregulate the expression of rRNA, thereby mediating the production of heterogeneous ribosomes and promoting viral protein translation (Goh et al., 2004). It is well known that HCV is one of the causes of hepatic cancer, which may be related to abnormal ribosome function leading to uncontrolled protein synthesis and increased demand for ribosome biosynthesis. A previous study reported that the matrix protein (M) encoded by Newcastle disease virus (NDV) at the beginning of infection is mainly localized in the nucleolus, and it may affect ribosome biogenesis and inhibit the synthesis of host proteins (Peeples et al., 1992). Presently, RPs that interact with the swine acute diarrhea syndrome coronavirus (SADS-CoV) M protein are quantified by liquid chromatography-mass spectrometry (LC–MS/MS) methods (Xu et al., 2022). This is consistent with the argument of Duan et al. on how M protein entry into the nucleus promotes RPL18 production found in NDV (Duan et al., 2022). Mouse L cells infected by VSV show inhibition of the synthesis of 45S rRNA precursors and their processing into 28S and 18S rRNA, which prevents ribosome assembly and RP synthesis (Jaye et al., 1980; Zan et al., 1990). With this similarity, the HIV Tat protein is known to be localized primarily in the nucleus and nucleolus. Interestingly, as Tat expression increases, cytoplasmic ribosome production decreases due to Tat’s interaction with the pro-fibronectin-U3 snoRNA complex, which interferes with the maturation of nucleolus precursor rRNA (Ponti et al., 2008). By using this mechanism, HIV regulates the host response, leading to apoptosis and shutting down protein synthesis in uninfected HIV cells (Ponti et al., 2008). In recent studies, researchers have found that influenza A virus (IAV) NS1 and the poliovirus 3C protease inhibit the transcription of rDNA in the nucleolus, while the infectious bronchitis virus (IBV) N protein can enter the nucleolus together with RPs to prevent the formation of new ribosomes and disrupt the cell cycle to interfere with translation in the host cell (Hiscox et al., 2001; You et al., 2005). However, the inhibition of ribosome biogenesis by this strategy is as adverse to virus proliferation as preventing the assembly of ribosomal subunits. Hence, the precise mechanism of these viruses needs to be further explored.

The host is a whole interconnected organism, and viral infections can affect the metabolic functions of the organism in various ways. Viruses and host cells are in a constant state of battle, and an increasing number of mechanisms and countermeasures are gradually being revealed that play an important role in maintaining an effective infection process and the stability of the internal environment of the host cell.

#### Differences in ribosomal components induced by viral infection

rRNA and RPs are the main components of ribosomes. Presently, we know that viral infection induces post-translational modifications of RPs, but it remains unknown whether rRNA changes occur due to viral infection. rRNAs are the main effectors of ribosome dynamics during mRNA decoding, peptide bond formation, and translation (Erales et al., 2017). In addition to being components of ribosomes, RPs play an important role in ribosome biogenesis, and they regulate many fundamental life processes, including cell cycle, cell proliferation, apoptosis, tumor development, genome integrity and development, and so on (Li, 2019; Miller et al., 2021).

Mutations, amplicon changes, and post-transcriptional modifications of rRNA alleles not only affect the fidelity of ribosomal translational mRNA, but are also a major aspect that contributes to ribosomal heterogeneity (Sloan et al., 2017; Fujii et al., 2018; Poitevin et al., 2020; Jansson et al., 2021; Gay et al., 2022). However, the effects of viral infection-induced changes in rRNA need further study. As mentioned above, IRES structures encoded by the viral genome bind to the 40S subunit to facilitate the translation of viral proteins. There is abundant evidence that encephalomyocarditis virus (EMCV), FMDV, hepatitis A virus (HAV), and HCV control the ribosome through the interaction of the IRES structure of genomic RNA with 18S rRNA in the 40S ribosomal subunit to translate viral proteins (Le et al., 1993, 1995). By using chemical detection and cryogenic electron microscopy (cryo-EM), the genome of HCV-infected cells was found to be complementary to the amplified segment 7 (ES7) base sequence of 18S rRNA (Malygin et al., 2013; Quade et al., 2015). Epstein–Barr virus (EBV), one of the DNA viruses, encodes the transcription factor Rta, which binds to rRNA in the 40S, 60S and 80S ribosomal subunits of nucleosomes and promotes the formation of heterogeneous ribosomes to preferentially translate the viral genome (Hwang et al., 2020). Such binding mechanisms remain to be explored in RNA virus-infected cells. Eukaryotic rRNAs include 18S rRNA, 28S rRNA, 5.8S rRNA, and 5S rRNA. Further studies are required to determine whether virus infection causes changes in rRNA mutation and modification.

Differences in post-translational modifications of RPs, such as phosphorylation, acylation, methylation, or glycosylation, contribute to ribosomal heterogeneity. Recent studies have shown that the deletion of RPs that constitute the subunits of the ribosome alters the level of translation of cellular proteins (Emmott et al., 2019; Luan et al., 2022). Different viral infections lead to various phosphorylation modifications of the ribosomal protein; this facilitates the expression of viral proteins (DiGiuseppe et al., 2020). Regarding phosphorylation of RACK1, researchers suggest that mutation of the phosphorylation site in the RACK1 protein to a negatively charged amino acid not only results in a conformational change in the 80S ribosomal subunit and a change in the conformation of the 40S ribosomal head, but it also promotes viral protein translation (Rollins et al., 2021). In summary, viral infection and modification of ribosomal components lead to heterogeneity of ribosomal function and promote the expression of viral proteins. However, the mechanisms by which heterogeneous ribosomes selectively translate viral proteins need to be further investigated.

#### Conformational changes in ribosomes induced by viral infection

Currently, it is known that the different translation strategies used by the viral genome to manipulate the ribosome cause changes in ribosomal conformation (Poitevin et al., 2020). However, it is not sufficient to conduct quantitative comparisons of ribosome structures, and comparisons of ribosome composition and studies of ribosome conformation are required (Poitevin et al., 2020). RPL40 promotes translation initiation in VSV, MV, and rabies virus (Lee et al., 2013). Moreover, one cannot help but suspect that during VSV infection, the conformation of the 60S subunit may change in response to the interaction of viral mRNA with RPL40 to allow the ribosome to preferentially translate the viral genome. By using cryo-EM, the spatial structure of the interaction between alphavirus mRNA and host ribosomal 18S rRNA was revealed, and this interaction resulted in a conformational change of rRNA extended segment 6 (ES6S) that is necessary for alphavirus translation (Toribio et al., 2016, 2018). In addition, it was found in HCV IRES translation initiation that the viral genome utilizes eIF2 or eIF5B to form a specific 48S complex conformation, which in turn recruits the 60S ribosomal large subunit to initiate protein translation (Brown et al., 2022). Thus, it was demonstrated that viral infection manipulates the ribosome and induces conformational changes in the ribosome to selectively translate viral mRNA.

To date, there are no reports that identify viral-induced heterogeneous ribosomal components. Therefore, it is crucial to conduct more studies on heterogeneous ribosomes to clarify the translation mechanism of viral proteins.

## Ribosomal proteins exert non-ribosomal functions during RNA virus infection

Viruses antagonize the antiviral immune response of the host and promote virus replication through complex interactions with the host. At present, several studies have shown that ribosomes have non-ribosomal functions in addition to translation after virus infection.

Ribosomal proteins positively regulate viral proliferation, mainly by promoting the replication, transcription, and assembly of viral genomes or by serving as viral receptors. The lymphocytic choriomeningitis virus (LCMV) Z protein interacts with the ribosomal protein RPLP0 and plays an important role in virion assembly (Pedersen, 1973; Borden et al., 1998). Moreover, the LCMV replication-transcription complex (RTC) contains the RPs eL10a and eS6, and these RPs facilitate replication of the viral genome. By interacting with RPs, the LCMV nucleoprotein (N) can inhibit the antiviral immune response of the host (Baird et al., 2012). Similarly, the 3′-extremity region of rabbit hemorrhagic disease virus (RHDV) interacts with RPS5 to promote replication of viral genes (Guo et al., 2020). Infectious bursal disease virus (IBDV) VP3 protein interacts with RPs to form ribosomal protein assembly complexes to promote viral genome replication (Ma et al., 2021). HIV-1 encodes Gag proteins that interact with RPL7 to facilitate the assembly of viral particles (Karnib et al., 2020). The efficient particle assembly of mouse mammary tumor virus (MMTV) depends on the interaction of Gag and RPL9 in the nucleolus of infected cells (Beyer et al., 2013). RPS2 (RPSA), as a ribosomal binding receptor, binds to the envelope glycoprotein E of DENV and yellow fever virus to promote viral infection (Zidane et al., 2012). After Semliki Forest virus (SFV) invades host cells, it needs the large ribosomal subunit to uncoat and release the viral genome (Wengler et al., 1992). Existing studies have shown that RPs can not only play an important role in the translation of the viral genome as a major component of the protein translation factory, but they can also contribute to viral life cycle progression.

Some RPs have antiviral functions. RPs interact with viral proteins to directly inhibit viral transcription or translation. During CSFV infection, the viral protein Npro interacts with uS10 and induces Npro degradation by the proteasome (Lv et al., 2017). Because TLR3 overexpression or uS10 knockdown does not promote CSFV replication, it was found that uS10 may indirectly regulate CSFV replication through TLR3 (Lv et al., 2017). Some RPs also activate antiviral signaling pathways. The ribosomal protein RPSA interacts with FMDV structural proteins to inhibit the activation of the mitogen-activated protein kinase (MAPK) pathway, thereby inhibiting viral genome replication (Zhu et al., 2020). Finally, RPS3, as an example, not only plays a role in apoptosis and DNA repair, but it also acts as a novel antiviral factor to inhibit CSFV proliferation by increasing the secretion of antiviral cytokines (Zhao et al., 2022).

In summary, the biological significance of many RPs in the infection of different viruses is gradually being revealed. In recent years, the interaction between viral and host proteins has been analyzed by high-throughput quantitative mass spectrometry where over 50 RPs interact with SFV capsid proteins (Contu et al., 2021). However, the specific functions need further study. Similarly, a proteomic approach has been used to explore the interactions between the encoded viral proteins and host proteins following IBDV infection (Ma et al., 2021). Therefore, the above finding demonstrates the feasibility of this approach for determining the role of viral proteins following viral invasion of the host. Currently, two-dimensional gel electrophoresis and mass spectrometry are commonly used in proteomics, and the abovementioned studies also suggest that the use of proteomic techniques, ribosome analysis, and bioinformatics to analyze ribosomal components has far-reaching potential.

## Summary and prospects

Ribosomes are protein synthesis factories not only for eukaryotic mRNA but also for viral mRNA. After the virus infects the host cell, it needs to manipulate the ribosome to complete the translation of viral protein. The control of the ribosome by viruses involves two main aspects. First, viruses shut down the ribosome to translate host mRNA. For example, some viral proteins regulate the compartmentalization of organelles or recruit translation initiation factors to facilitate the assembly of the translation machinery and viral protein translation. To inhibit host mRNA translation by the ribosome, viruses also promote the assembly of large/small ribosomal subunits by regulating the secondary structure of viral mRNAs. Second, viruses manipulate the ribosome to complete viral protein translation. The viral mRNA secondary structure not only influences the assembly rate of the large and small subunits of the ribosome, but it also regulates how ribosomes move on the viral mRNA during different translation stages. Viral infection also induces changes in the composition and conformation of ribosomes, resulting in the formation of heterogeneous ribosomes. Therefore, analyzing the mechanism of viral control of ribosomes and studying the properties and assembly mechanisms of heterogeneous ribosomes required for viral protein translation will help us to fully understand the mechanism of viral protein translation.

With the development of science and technology, especially proteomics and cryo-EM, the structure of eukaryotic ribosomes has been gradually revealed. The crystal structure of the human 80S ribosome and the crystal structure of the yeast 90S ribosomal precursor has been reported, thereby opening a new door for designing small-molecule drugs targeting the ribosome (Khatter et al., 2015; Sun et al., 2017). Some recent studies have shown that Alzheimer’s disease, Parkinson’s disease, tumorigenesis, cancer metastasis, and other diseases are associated with the abnormal ribosomal function (Ebright et al., 2020; Elhamamsy et al., 2022; Stein et al., 2022). The necessity for correct assembly of ribosomes for hematopoietic stem cell regeneration and the involvement of the ribosomal protein RPL39L in spermatogenesis also reflect the importance of ribosomes for maintaining body homeostasis and genetic stability (Lv et al., 2021; Zou and Qi, 2021). Because these diseases are dependent on abnormal ribosomal function, we wondered whether this “butterfly effect” could be cured by developing drugs that target ribosomes. The antibiotic tetracenomycin blocks bacterial protein synthesis by targeting bacterial ribosomes, and it is precise because of this targeting that the emergence of bacterial resistance can be effectively avoided (Osterman et al., 2020). This discovery suggests a promising future for the development of drugs targeting heterogeneous ribosomes. The heterogeneity of ribosomal components in Kaposi’s sarcoma-associated herpesvirus infected cells has been analyzed using quantitative proteomics (Murphy et al., 2022). The deletion of ribosomal biogenesis factor BUD23 was found to significantly inhibit the replication of viral particles (Murphy et al., 2022). This suggests that the inhibition of new heterogeneous ribosome production after viral infection of host cells can effectively kill viral particles in the organism. Therefore, the identification and structural analysis of the heterogeneous ribosomal components required for viral protein translation will contribute to a comprehensive understanding of the mechanism of viral protein synthesis and open a new door for developing antiviral drugs in the future. It also provides new strategies for optimizing vaccines and raises new questions for RNA vaccines or drugs, that is, whether related drugs or vaccines after entering the body will have corresponding adverse reactions on ribosomes.

## Author contributions

GL and JZ: conceptualization and review and editing. XW and JZ: writing original draft preparation. GL: supervision and project administration. DZ and XW: visualization. All authors have read and agreed to the published version of the manuscript.

## Funding

This study was sponsored by the Natural Science Foundation of Shanghai (22ZR1476300), the National Natural Science Foundation of China (32000109 and 32172832), the Shanghai Sailing Program (20YF1457700), Shanghai Municipal Science and Technology Major Project (ZD2021CY001), and the Central Public-interest Scientific Institution Basal Research Fund (2022JB01).

## Conflict of interest

The authors declare that the research was conducted in the absence of any commercial or financial relationships that could be construed as a potential conflict of interest.

## Publisher’s note

All claims expressed in this article are solely those of the authors and do not necessarily represent those of their affiliated organizations, or those of the publisher, the editors and the reviewers. Any product that may be evaluated in this article, or claim that may be made by its manufacturer, is not guaranteed or endorsed by the publisher.
